# Cassette-Based Automated Production of 2-Deoxy-2-[^18^F]fluorocellobiose on the Trasis AllInOne with Undetectable [^18^F]FDG Contamination

**DOI:** 10.3390/molecules31081260

**Published:** 2026-04-10

**Authors:** Falguni Basuli, Jianfeng Shi, Swati Shah, Jianhao Lai, Dima A. Hammoud, Rolf E. Swenson

**Affiliations:** 1 Chemistry and Synthesis Center, National Heart, Lung, and Blood Institute, National Institutes of Health, Bethesda, MD 20892, USA; jianfeng.shi@nih.gov (J.S.); rolf.swenson@nih.gov (R.E.S.); 2 Center for Infectious Disease Imaging, Radiology and Imaging Sciences, Clinical Center (CC), National Institutes of Health, Bethesda, MD 20892, USA; swati.shah@nih.gov (S.S.); jianhao.lai@nih.gov (J.L.); hammoudd@cc.nih.gov (D.A.H.)

**Keywords:** fluorine-18, automated, Trasis AIO, FDG, cellobiose, enzyme, fungal infection, PET

## Abstract

The global rise in the incidence and severity of invasive fungal infections, particularly among immunocompromised and immunodeficient patients, has created an urgent need for rapid and accurate diagnostic techniques. Therefore, fungal-specific positron emission tomography imaging agents are increasingly in demand, as they offer the potential for early-stage detection of fungal infections. Recently, 2-deoxy-2-[^18^F]fluorocellobiose ([^18^F]FCB), a fluorine-18-labeled analog of cellobiose that is selectively metabolized by fungal pathogens possessing cellulose-degrading mechanisms (cellulolytic), was developed for the targeted imaging of Aspergillus infections. However, the final [^18^F]FCB contained less than 2% unreacted 2-deoxy-2-[^18^F]fluoroglucose ([^18^F]FDG), which can potentially interfere with image interpretation. Accordingly, this study aims to eliminate residual [^18^F]FDG from the final product by enzymatically converting it to [^18^F]FDG-6-phosphate through hexokinase-mediated phosphorylation. A Trasis AllInOne (Trasis AIO) module was used to automate the radiolabeling procedure. The reagent vials contain [^18^F]FDG, glucose-1-phosphate, cellobiose phosphorylase, adenosine triphosphate (ATP), and hexokinase. A Sep-Pak cartridge was used to purify the tracer. The overall radiochemical yield was 45–50% (*n* = 3, decay-corrected) in a 40 min synthesis time, with a radiochemical purity of >99% (no detectable [^18^F]FDG). This is a highly reliable protocol to produce current good manufacturing practice (cGMP)-compliant [^18^F]FCB for clinical PET imaging.

## 1. Introduction

Fungi are remarkably adaptable organisms that can colonize diverse environments, including soil, air, water, and living hosts such as plants, animals, and humans [1,2,3]. They can cause invasive fungal infections (IFIs), typically triggered by pathogenic yeasts or molds, which pose serious risks [4,5]. These infections are more common in individuals with compromised immune systems [6,7,8]. Among these, *Aspergillus* species are major pathogens responsible for IFIs in hospital settings, particularly in intensive care unit (ICU) patients [9,10]. Early detection of such infections is critical for initiating timely and effective treatment to reduce the high associated mortality rates. However, conventional imaging techniques such as X-rays, computed tomography (CT), magnetic resonance imaging (MRI), and ultrasound lack the specificity and sensitivity required to identify IFIs in their early stages [8,11]. Culture-based diagnostic methods, while definitive, often require invasive sample collection and may take several days to yield results, thus delaying the start of appropriate therapy [12,13,14]. These limitations emphasize the urgent need for more rapid, sensitive, and non-invasive diagnostic approaches.

Molecular imaging in preclinical animal models can detect pathological changes before the onset of clinical signs, allowing experiments to be performed earlier and reducing mortality while still providing survival data. Moreover, the same animals can be used longitudinally, allowing baseline data collection before infection and intervention, thereby reducing the number of animals needed and enabling more robust comparisons [15,16]. Because positron emission tomography (PET) offers superior spatial resolution and sensitivity compared to SPECT, PET is widely accepted as the imaging modality of choice for early disease detection in various infections [17,18,19]. The gold-standard metabolically trapped PET tracer, 2-deoxy-2-[^18^F]fluoroglucose ([^18^F]FDG), which has been successfully evaluated in oncology and neurology, has also been investigated for the detection of IFI [20,21,22]. However, its use is limited due to its inability to distinguish between IFI, sterile inflammation, bacterial infections, and other etiologies, including cancer [23,24,25]. Our group recently developed an enzymatic method to produce 2-deoxy-2-[^18^F]fluorocellobiose ([^18^F]FCB), a fluorine-18-labeled derivative of cellobiose, a disaccharide selectively metabolized by β-glucosidase (βGL) producing fungi such as *Aspergillus fumigatus* (*A. fumigatus*) [26]. Just as cellobiose is metabolized by those cellulolytic molds into two glucose molecules, [^18^F]FCB is metabolized by those fungi into one glucose and one [^18^F]FDG unit. The latter is subsequently taken up and retained within the fungal cells, thus providing pathogen-specific imaging of those fungal infections [26]. In mammalian hosts and other bacterial infection models, on the other hand, β-glucosidases are not produced and [^18^F]FCB is instead renally excreted as an intact molecule, as evidenced by PET images of healthy animals.

The radiolabeling to produce [^18^F]FCB was initially performed manually. However, later, for clinical use, an automated synthesis process was developed [27] that streamlines PET radiopharmaceutical production, minimizes operator-dependent variability, and improves reproducibility [28,29,30]. In both the manual and automated productions, however, the final [^18^F]FCB solution contained <2% unreacted [^18^F]FDG. This was problematic because although [^18^F]FCB PET images showed minimal background signal, we still observed a persistent cardiac signal in the majority of mice. We surmised this signal to be due to the presence of residual [^18^F]FDG in the tracer solution, as this occurred in healthy controls and not just infected mice. This is significant because invasive aspergillosis often affects the lungs, and delineating specific uptake in lung lesions in the vicinity of the heart could be limited. Also, because the detection of fungal infection using [^18^F]FCB relies on its ability to break down to [^18^F]FDG, the presence of exogenous [^18^F]FDG, even in trace amounts, may lead to less confidence in the interpretation of imaging results in patients. This is especially important in suspected myocardial or heart valve infection with cellulolytic molds, resulting in myocarditis or endocarditis in those organs [31,32]. In the absence of exogenous [^18^F]FDG, any uptake would be produced by [^18^F]FCB metabolism with fungal βGLs, thus increasing diagnostic certainty. A low background signal in the highly metabolically active brain is also clinically relevant since high [^18^F]FDG uptake in normal neurons could potentially mask small infectious foci in the cortical gray matter or in the basal ganglia [33].

Therefore, preparation of [^18^F]FCB without any detectable contamination of [^18^F]FDG was pursued and is reported in this study. Briefly, after initial incubation of the reaction mixture ([^18^F]FDG, Glc-1P and CBP) to produce [^18^F]FCB, the remaining unreacted [^18^F]FDG was converted to [^18^F]FDG-6-phosphate by adding ATP/hexokinase and incubating for an additional 5 min. The resulting reaction mixture was purified using the same cartridge-based purification method described in the literature [26,27]. Additionally, a fully automated cassette-based method has been developed to prepare [^18^F]FCB using the Trasis AllInOne (Trasis AIO) module. The simplicity of this method will allow large-scale routine production of the tracers in a current good manufacturing practice (cGMP)-compliant laboratory.

## 2. Results and Discussion

### 2.1. Chemistry

**Scheme 1:**
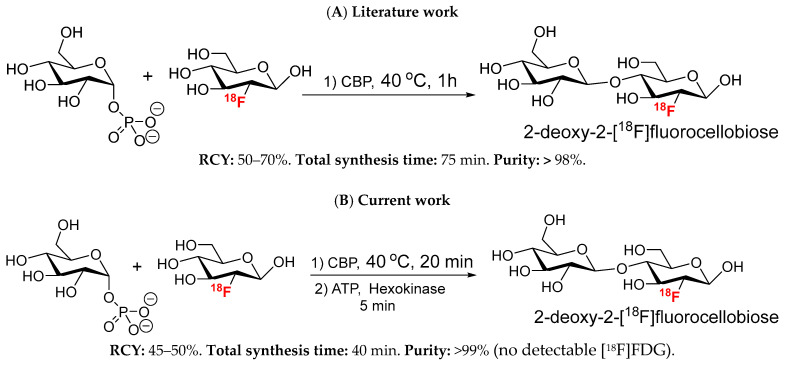
Synthesis of [^18^F]FCB: (**A**) literature method; (**B**) current method.

Previously, our group developed an enzymatic method (Scheme 1A) for synthesizing 2-deoxy-2-[^18^F]fluorocellobiose ([^18^F]FCB) from commercially available 2-deoxy-2-[^18^F]fluoroglucose ([^18^F]FDG) [26,27]. Briefly, in a typical procedure, 100 mCi of [^18^F]FDG (~1.2 mL of saline), 37 mg of glucose-1-phosphate (Glc-1P) (100 μL of water), 1 M HEPES buffer pH 7.6 (100 μL) and CBP (1.5 mg in 100 μL 50 mM Tris-HCl, 500 mM NaCl, 10% Glycerol, pH 8.0, final concentration 1 mg/mL) were incubated for 1 h at 40 °C followed by purification using an FDG purification cartridge. The method was also automated using the Trasis AIO module. The overall yield of the reaction was 50–70% (*n* = 6, decay-corrected) in a 75 min synthesis time.

**Figure 1:**
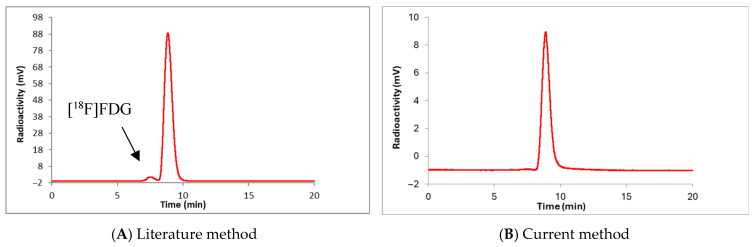
Radio HPLC chromatogram of [^18^F]FCB. HPLC conditions: Agilent AdvanceBio glycan (250 × 4.6 mm), 2.7 μm; eluent, 68–62% B in A for 15 min; flow rate, 0.5 mL/min. B: acetonitrile; A: 50 mM ammonium formate.

**Figure 2:**
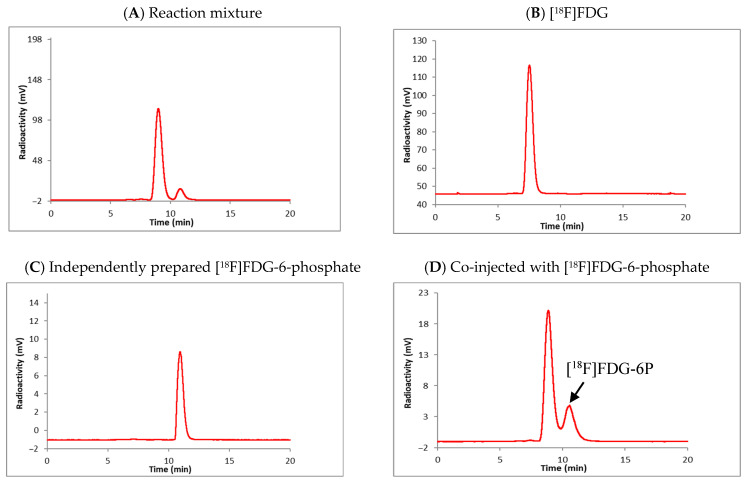
HPLC of (**A**) reaction mixture of [^18^F]FCB; (**B**) [^18^F]FDG; (**C**) [^18^F]FDG-6P independently prepared; (**D**) [^18^F]FCB co-injected with [^18^F]FDG-6P. HPLC conditions as mentioned in Figure 1.

The method is highly reliable and provides an excellent radiochemical yield. Nonetheless, the final [^18^F]FCB solution contains <2% [^18^F]FDG. A representative HPLC chromatogram of [^18^F]FCB is shown in Figure 1A, indicating the presence of a minor radioactive peak corresponding to [^18^F]FDG. This residual [^18^F]FDG can accumulate nonspecifically in areas of inflammation or alternative infectious foci, potentially producing false-positive signals. Therefore, removal of unreacted [^18^F]FDG from the final product would improve the certainty of diagnosis since [^18^F]FCB would be the only source for [^18^F]FDG, which is through fungal metabolism. While the FDG cartridge purification method efficiently eliminates the enzyme (CBP), it does not separate unreacted [^18^F]FDG from [^18^F]FCB. As a result, achieving a radiochemical purity of >98% relies on the near-quantitative enzymatic conversion of [^18^F]FDG to [^18^F]FCB during the initial incubation process. Based on our previous observations, an enzyme concentration of 1 mg/mL is required to achieve near-quantitative conversion to produce [^18^F]FCB [26]. However, because the volume of commercially supplied [^18^F]FDG varies between production batches, larger volumes require proportionally higher amounts of enzyme, making the process both costly and potentially inefficient. To address this limitation, an alternative purification strategy is explored in this study to remove residual [^18^F]FDG. We hypothesized that phosphorylation of [^18^F]FDG would permit more selective and effective purification of the final [^18^F]FCB radiotracer using the previously employed FDG cartridge. Hexokinase is a regulatory enzyme in glycolysis, catalyzing the rate-limiting irreversible phosphorylation of glucose into glucose-6-phosphate. In this new strategy, after the enzymatic conversion of [^18^F]FDG to [^18^F]FCB, a mixture of ATP and hexokinase was added to the reaction mixture and incubated for an additional 5 min. The HPLC profile of the reaction mixture indicated no detectable [^18^F]FDG peak, which was observed with the earlier method (Figure 1A), and instead revealed a new peak after 10 min (Figure 2A). To identify this new peak, [^18^F]FDG-6-phosphate was prepared independently, following a literature method [34], and used for co-elution analysis. Briefly, a mixture of ATP (10 mg) and hexokinase (5 mg) in 100 μL HEPES buffer (pH 7.6) was added to the [^18^F]FDG solution (30 mCi in 1 mL of saline), and the mixture was incubated at 40 °C for 5 min. The progress of the reaction was monitored by HPLC, showing complete conversion of [^18^F]FDG to [^18^F]FDG-6-phosphate within 5 min (Figure 2B,C). Co-elution of this independently prepared [^18^F]FDG-6-phosphate with the peak after 10 min in the reaction mixture of [^18^F]FCB confirmed the identity of the peak (Figure 2D) as [^18^F]FDG-6-phosphate. As anticipated, [^18^F]FDG-6-phosphate was retained in the FDG purification cartridge, and pure [^18^F]FCB (Figure 1B) was eluted with normal saline with a radiochemical purity of >99% (Figure 1B, no detectable [^18^F]FDG). This is due to the negatively charged phosphate group in [^18^F]FDG-6-phosphate strongly binding with the anion-exchange resin in the cartridge. Due to this modification to the synthetic procedure, the current method no longer necessitates waiting for near-quantitative conversion, as the unreacted [^18^F]FDG is converted to [^18^F]FDG-6-phosphate for efficient removal using the FDG cartridge; as a result, the initial incubation period was shortened to 20 min (vs. 1 h). Therefore, although the protocol includes an additional step, the overall reaction time is reduced by 35 min. Additionally, the same cartridge purification method effectively removes hexokinase (Supplementary Materials, Figure S1).

**Figure 3:**
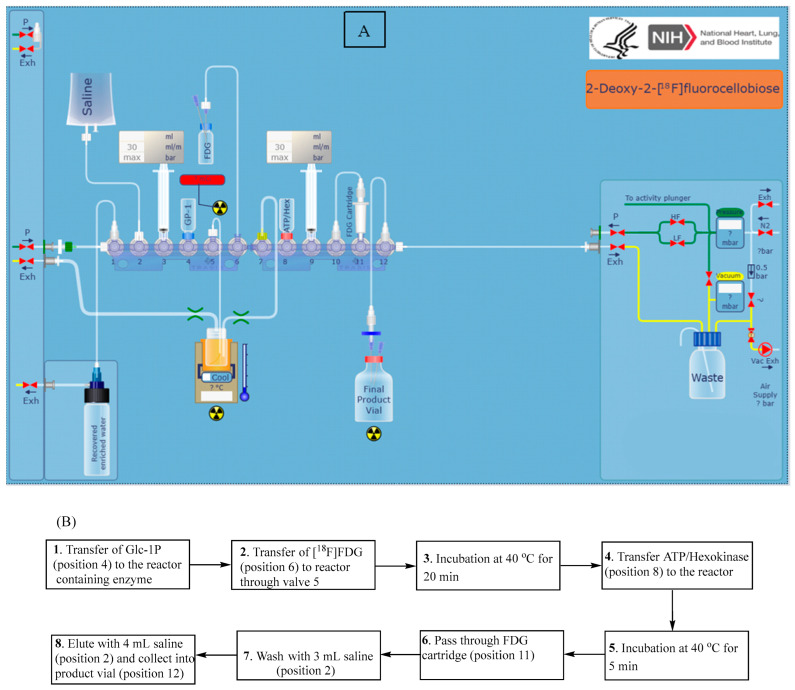
Automated synthesis of [^18^F]FCB. (**A**) Schematic diagram of the Trasis AIO module; (**B**) key steps for automatic synthesis of [^18^F]FCB.

After manual optimization, a fully automated cassette-based synthesis of [^18^F]FCB was performed using the Trasis AIO module. A schematic diagram of the automated synthesis, along with the key steps, is displayed in Figure 3. Glc-1P (37 mg in 100 μL of water) from Position 4 was transferred to the reaction vial containing CBP (1 mg in 100 μL of 50 mM Tris-HCl buffer, pH 8.0), followed by [^18^F]FDG (100–150 mCi in ~2 mL of saline). The reaction mixture was incubated for 20 min at 40 °C. ATP/hexokinase (Position 8) was transferred to the reactor and incubated for 5 min at the same temperature. The reaction mixture was passed through an FDG cartridge (Position 11) to retain [^18^F]FCB and [^18^F]FDG-6-phosphate on the cartridge. The cartridge was washed with 3 mL of saline, followed by the elution of only [^18^F]FCB ([^18^F]FDG-6-phosphate remained on the cartridge) with 4 mL of saline through a sterile filter (Vented Millex^®^ 0.22 μm, MilliporeSigma, St. Louis, MO, USA) into the product vial at Position 12.

**Figure 4:**
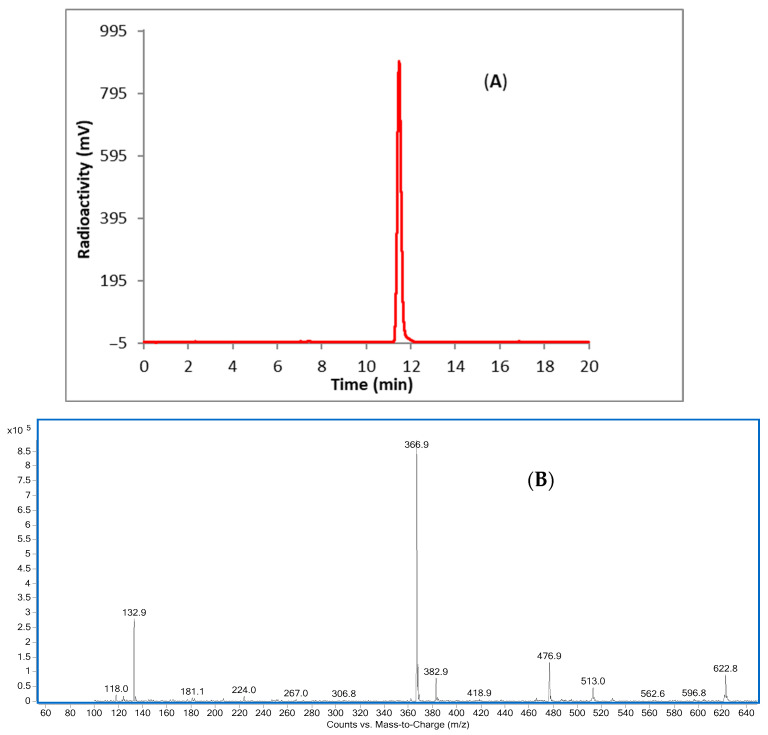
LCMS of [^18^F]FCB, co-injected with non-radioactive standard FCB. (**A**) Radio detector; (**B**) ESI Mass Spec calculated for C_12_H_21_FO_10_Na 367.10 found 366.9 (M + Na). HPLC conditions: column, XBridge amide (4.6 mm × 150 mm, 3.5 μm); eluent, 90–50% B in 16 min; (**A**) = 95:5 water:acetonitrile + 0.1% NH_4_OH; (**B**) = 5:95 water:acetonitrile + 0.1% NH_4_OH; flow rate, 0.5 mL/min.

The overall yield of the reaction was 45–50% (*n* = 3, decay-corrected) in a 40 min synthesis time with a radiochemical purity of >99%. Although the yield is high, it is slightly lower than that reported in the literature (50–70%), where <2% residual [^18^F]FDG is present in the final [^18^F]FCB dose. This discrepancy is likely due to the shorter initial incubation time used in the present method (20 min vs. 1 h). As noted earlier, the literature protocol relies on near-quantitative conversion of [^18^F]FDG to [^18^F]FCB and therefore requires a longer incubation period. The identity of the product was confirmed using liquid chromatography–mass spectrometry (LCMS) by co-eluting with the non-radioactive standard and matching the mass peak with the radiation peak (Figure 4). In a typical radiolabeling starting with 150 mCi of [^18^F]FDG, 55 mCi of the product was obtained in 40 min (47% RCY, decay-corrected). Residual Glc-1P, CBP, hexokinase, and ATP in the final dose of [^18^F]FCB were analyzed by HPLC under different conditions (Supplementary Materials, Figures S1–S3). No detectable peaks were observed for any of these reagents, indicating that their concentrations were below the detection limits of the analytical methods. The results are summarized in Table 1. [^18^F]FCB showed excellent stability in whole human serum at 37 °C (Supplementary Materials, Figure S4).

### 2.2. PET Imaging in Healthy Mice

**Figure 5:**
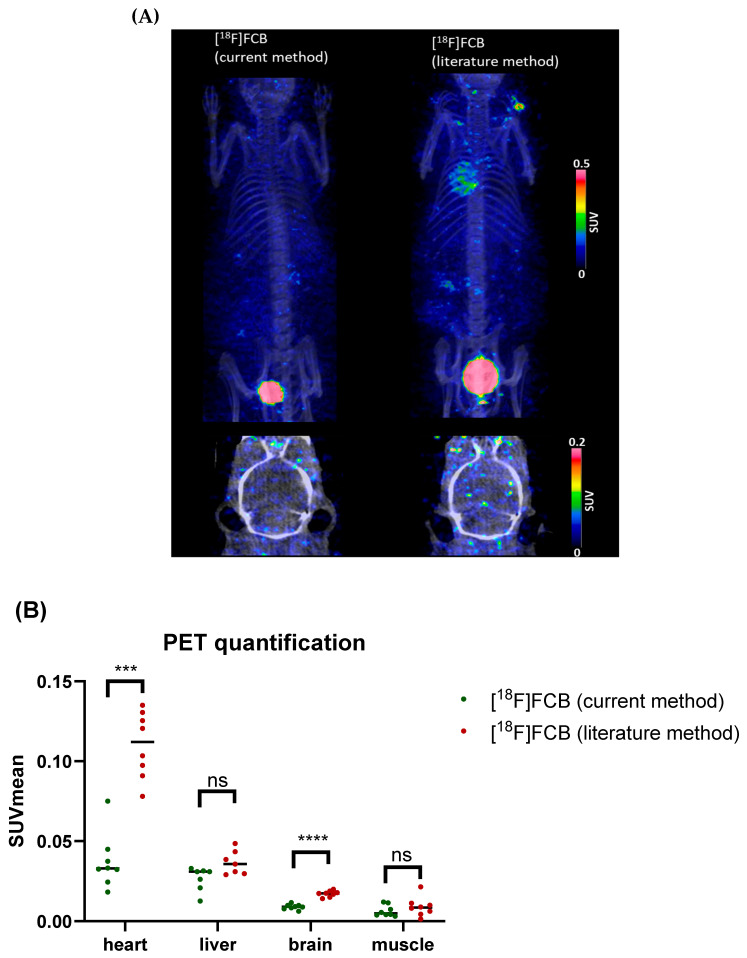
Static PET/CT imaging of healthy mice injected with either [^18^F]FCB prepared by the current method or the literature method. (**A**) Maximum intensity projection (MIP) images of representative whole-body PET/CT scans and representative axial PET/CT images of the brain. (**B**) PET quantification of tracer uptakes in the heart (*n* = 8), liver (*n* = 7), brain (*n* = 8), and muscle (*n* = 8). Statistical analysis using multiple paired *t*-tests with Holm–Sidak correction for multiple comparisons was performed; *** *p* < 0.001 **** *p* < 0.0001. ns, not significant.

Healthy mouse cohorts were injected with [^18^F]FCB (Figure 5), prepared following either the current method (no [^18^F]FDG) or the previous method, resulting in <2% residual [^18^F]FDG. The imaging was performed in two separate sessions, with each animal receiving either one of the tracers during each session. At 2 h post tracer injection (to allow for vascular clearance), the animals underwent PET/CT imaging. When animals received >98% pure [^18^F]FCB produced by the literature method, their myocardial and brain uptake were significantly higher than when they received >99% pure [^18^F]FCB produced by the current method (myocardium: 0.1102 ± 0.0206 vs. 0.0373 ± 0.0172, *p* < 0.001; brain: 0.0172 ± 0.0018 vs. 0.0090 ± 0.0016, *p* < 0.0001 multiple paired *t*-tests with correction for multiple comparisons). At the same time, there were no significant differences in muscle uptake between the prior and current tracer preparations, suggesting residual [^18^F]FDG is mainly noted in highly metabolically active organs (Figure 5B), which rely mainly on glucose as an energy source [35,36]. Interestingly, even though not statistically significant, there was generally lower uptake in the liver with >99% pure [^18^F]FCB produced by the current method compared to >98% pure [^18^F]FCB produced by the literature method. A decrease in the background signal of the liver could potentially improve the certainty of diagnosis when liver fungal infections are suspected. We thus believe that the efficient purification of [^18^F]FCB described in this study will eventually allow for better detection of infections by cellulolytic molds in the brain myocardium, liver, and potentially other organs.

## 3. Materials and Methods

Cellobiose phosphorylase (CBP) was obtained from GenScript (Piscataway, NJ, USA). All other chemicals were purchased from MilliporeSigma (St. Louis, MO, USA) and used without further purification. [^18^F]FDG was obtained from Cardinal Health (Silver Spring, MD, USA). The FDG purification cartridge (Base Hydrolysis, KT-100) was obtained from ISOFLEX (San Francisco, CA, USA) and conditioned with 12 mL of ethanol followed by 30 mL of water. Acrodisc^®^ Sterile Syringe Filters (5 μm) with Supor^®^ Membrane were purchased from VWR (Radnor, PA, USA). The 1290 Infinity II evaporative light scattering detector (ELSD) was obtained from Agilent Technologies (Santa Clara, CA, USA) and Waters (Milford, MA, USA). The SiELC Newcorm B column was purchased from SIELC Technologies (Wheeling, IL, USA). Other columns and Sep-Pak cartridges used in this synthesis were obtained from Agilent Technologies (Santa Clara, CA, USA) and Waters (Milford, MA, USA), respectively. High-performance liquid chromatography (HPLC) analyses were performed on an Agilent 1200 Series instrument equipped with a multi-wavelength UV detector connected in series with a Bioscan flow count radio detector (Eckert & Ziegler, B-FC-3500 diode, Wilmington, MA, USA). LC-MS/MS analysis was performed on an Agilent 6460C triple quadrupole mass spectrometer with an ESI source and a flow count radio detector (Eckert & Ziegler, B-FC-3500 diode, Wilmington, MA, USA). The LC inlet was an Agilent 1200 series chromatographic system equipped with a 1260 binary pump, 1290 thermostatted column compartment, and 1260 high-performance autosampler (Agilent Technologies, Santa Clara, CA, USA). Instrument control and data processing were performed using Agilent’s Mass Hunter Software (Version B.07.01).

### 3.1. HPLC Conditions

#### 3.1.1. Detection of [^18^F]FDG, [^18^F]FCB, [^18^F]FDG-6-Phosphate, and Stability Study

Column, Agilent AdvanceBio glycan (250 × 4.6 mm), 2.7 μm; eluent, 68–62% B in A for 15 min; flow rate, 0.5 mL/min; B = acetonitrile; A = 50 mM ammonium formate.

#### 3.1.2. Detection of CBP and Hexokinase

Column, TSKgel SuperSW3000 (4.6 mm ID × 30 cm, 4 μm); eluent, 0.1 M sodium phosphate, 0.1 M sodium sulfate, 0.05% sodium azide, 10% iso-propyl alcohol (pH 6.8); flow rate, 0.35 mL/min; UV detector at 280 nm.

#### 3.1.3. Detection of ATP

Column, Agilent XDB C-18 (4.6 × 150 mm, 5 μm); eluent, 5–100% B in A for 15 min: A = 0.1 M potassium phosphate monobasic (+4 mM potassium bisulfate) in water; B = 70% A and 30% methanol; flow rate, 0.5 mL/min; UV detector is at 254 nm.

#### 3.1.4. Detection of Glc-1P

Column, SiELC Newcorm B (4.6 × 150 mm, 5 μm, 100 A); eluent, 10% B acetonitrile in water; flow rate, 1 mL/min; ELSD, evaporator temperature and nebulizer temperature, 40 °C, gas flow, 1.6 SLM.

### 3.2. Synthesis of [^18^F]FCB

#### 3.2.1. Manual Synthesis

A total of 100 mCi (~2 mL of saline) of [^18^F]FDG, 37 mg of glucose-1 phosphate (100 μL of water), and CBP (1 mg in 100 μL of 50 mM Tris-HCl buffer, pH 8.0) were incubated for 20 min at 40 °C followed by the addition of a mixture of ATP (10 mg in 20 μL of water) and hexokinase (5 mg in 20 μL of water) in 400 μL HEPES buffer pH 8. The mixture was incubated for an additional 5 min at 40 °C and slowly passed through the FDG purification cartridge. The cartridge was flushed with 3 mL of saline. The product was eluted with 4 mL of saline to obtain [^18^F]FCB. The purity of the product was confirmed by HPLC (Figure 1B).

#### 3.2.2. Fully Automated Synthesis Performed Using the Trasis AIO Module

The reagent vials contained the following: (a) [^18^F]FDG (50–150 mCi in 1–2 mL of saline); (b) Glc-1P (37 mg in 100 μL of water) in Position 4; (c) ATP (10 mg in 20 μL of water) and hexokinase (5 mg in 20 μL of water) in 400 μL HEPES buffer pH 8 (Position 8); and (d) CBP (1 mg in 100 μL of 50 mM Tris-HCl buffer, pH 8.0) in the reactor vial. Glc-1P from Position 4 was transferred to the reactor under vacuum. [^18^F]FDG solution was directly transferred to the reactor. The reaction mixture was incubated for 20 min at 40 °C. ATP/hexokinase (Position 8) was transferred to the reactor and incubated for 5 min at 40 °C. The manifold line was washed with 2 mL of saline twice (Position 2). The reaction mixture was passed through an FDG cartridge (Position 11) to retain [^18^F]FCB on the cartridge. The cartridge was washed with 3 mL of saline, followed by the elution of [^18^F]FCB with 4 mL of saline through a sterile filter (Vented Millex^®^ 0.22 μm) into the product vial at Position 12. The identity and purity of the product were confirmed by LCMS (Figure 4).

#### 3.2.3. Synthesis of [^18^F]FDG-6-Phosphate

[^18^F]FDG-6-phosphate was prepared according to the literature method [34]. ATP (10 mg) and hexokinase (5 mg) in 100 μL HEPES buffer (pH 7.6) were added to the solution of [^18^F]FDG (30 mCi, 1 mL of saline), and the mixture was incubated at 40 °C for 5 min. The quality of the product was tested by HPLC (Figure 2C). This solution was directly used for the identification of [^18^F]FDG-6-phosphate produced in the reaction mixture of [^18^F]FCB.

### 3.3. Stability Study

The stability of [^18^F]FCB was assessed by incubating 0.8 mCi in 100 μL with 500 μL of whole human serum at 37 °C. Every hour, a 5 μL aliquot was removed and directly analyzed by HPLC (Supplementary Materials, Figure S4).

### 3.4. PET Imaging in Mice and Data Analysis

All experimental procedures, including handling and care of the animals, were approved by the Animal Care and Use Committee of the CC at NIH and performed following relevant NIH policies (animal study protocol: DRD 25-01; original approval date: 11 January 2019; most recent re-approval date: 28 February 2025). All mice were housed in pre-sterilized filter-topped cages and given access to food and water.

PET imaging was performed in healthy female CD-1 mice aged 5–7 weeks (Charles River, Charleston, SC, USA). The mice were anesthetized with isoflurane before tracer injection. [^18^F]FCB was injected in the mice (*n* = 8) intravenously through the tail vein (~9 MBq). Two imaging sessions were performed. In the first session, 4 mice received >98% pure [^18^F]FCB produced by the literature method, and 4 mice received >99% pure [^18^F]FCB produced by the current method. In the second session, the same animals were reimaged, with reversal of the injection types. As such, all eight animals received one injection of >98% and one injection of >99% pure [^18^F]FCB. PET imaging was performed in these mice, 2 h after tracer injection, using the NanoScan PET/CT (Mediso, Budapest, Hungary). The images were reconstructed using Nucline NanoScan 3.04 software (Mediso). Volumes of interest (VOIs) were drawn over the tissues of interest based on the CT images and were analyzed using MIM 7.3.3 (MIM Software Inc., Beachwood, OH, USA) to calculate mean standardized uptake values (SUVmean) in different tissues (heart, liver, brain, and muscle). Normality was assessed for the datasets from each organ. Based on this, multiple paired *t*-tests were performed. Resulting *p*-values were adjusted for multiple comparisons across organs using the Holm–Sidak correction. No a priori exclusion criteria were set; outlier identification for all datasets was still performed using the Grubbs test, and one outlier was identified and excluded from liver SUVmean data. All statistical analyses were performed using GraphPad Prism 10.

## 4. Conclusions

In this study, we developed an improved method for synthesizing >99% with no detectable [^18^F]FDG pure [^18^F]FCB by effectively removing any residual unreacted [^18^F]FDG. This was achieved by converting unreacted [^18^F]FDG to [^18^F]FDG-6P using hexokinase and ATP, followed by the efficient removal of the latter through a cartridge-based purification method. Although two additional reagents (hexokinase and ATP) were introduced in the current procedure, the total reaction time was reduced while maintaining a high radiochemical yield. Moreover, no additional purification steps were required to remove these extra reagents. The procedure was first validated manually and transferred to a disposable cassette-based automated radiosynthesis on the Trasis AIO to improve reproducibility and ensure compliance with cGMP standards. The RCY was 45–50% with a radiochemical purity of >99%. In a typical radiolabeling starting with 150 mCi of [^18^F]FDG, 55 mCi of the product was obtained in 40 min (47% RCY, decay-corrected). The efficient purification of [^18^F]FCB described in this study may facilitate enhanced detection of infections caused by cellulolytic molds in the brain, myocardium, liver, and potentially other organs.

## Figures and Tables

**Table 1:** HPLC detection of residual reagents in the final dose of [^18^F]FCB.

Reagents	Column ^a^	Detector	Detection Limit (ppm)	Amount in [^18^F]FCB (ppm)
Glc-1P	SiELC Newcorm B	ELSD	50	<50
CBP	TSKgel SuperSW3000	UV280	0.4	<0.4
hexokinase	TSKgel SuperSW3000	UV280	5	<5
ATP	Agilent XDB C-18	UV254	0.12	<0.12

^a^ Full HPLC conditions are listed in the Materials and Methods (Section 3).

## Data Availability

The datasets generated and/or analyzed during the current study are available from the corresponding author upon reasonable request.

## Supplementary Materials

The following supporting information can be downloaded at https://www.mdpi.com/article/10.3390/molecules31081260/s1. Figure S1: HPLC chromatogram (A) Hexokinase (detection limit 5 ppm); (B) CBP (detection limit 0.4 ppm); (C) [^18^F]FCB showing the absence of the peak for hexokinase (8 min) and CBP (7 min). HPLC conditions, Column, TSKgel SuperSW3000 (4.6 mm ID × 30 cm, 4 μm); eluent, 0.1 M sodium phosphate, 0.1 M sodium sulfate, 0.05% sodium azide, 10% iso-propyl alcohol (pH 6.8), flow rate 0.35 mL/min; UV detector at 280 nm. Figure S2: HPLC chromatogram (A) ATP (detection limit 0.12 ppm); (B) [^18^F]FCB showing the absence of the peak for ATP (~8 min). HPLC conditions, Column, Agilent XDB C-18 (4.6 × 150 mm, 5 μm); eluent, 5–100% B in A for 15 min, A = 0.1 M Potassium phosphate monobasic (+4 mM Potassium bisulfate) in water, B = 70% A and 30% methanol, flow rate 0.5 mL/min, UV detector at 254 nm. Figure S3: HPLC chromatogram (A) Glc-1P (detection limit 50 ppm); (B) [^18^F]FCB showing the absence of the peak for Glc-1P (15:00 min). HPLC conditions, Column, SiELC Newcorm B (4.6 × 150 mm, 5 μm, 100 A); eluent, 10% B acetonitrile in water, flow rate 1 mL/min; ELSD, evaporator temperature and nebulizer temperature 40 °C, gas flow 1.6 SLM. Figure S4: Stability in whole human serum (A) 0 h; (B) 4 h. Figure S5: Maximum intensity projections of PET/CT scans obtained in the same animals using both tracer preparations (current method and literature method) in two different settings.

## References

[1] Naranjo-Ortiz M.A., Gabaldón T. (2019). Fungal evolution: Diversity, taxonomy and phylogeny of the Fungi. Biol. Rev..

[2] Boddy L., Hiscox J. (2016). Fungal Ecology: Principles and Mechanisms of Colonization and Competition by Saprotrophic Fungi. Microbiol. Spectr..

[3] Corrêa-Junior D., Zamith-Miranda D., Frases S., Nosanchuk J.D. (2026). From the Ground to the Clinic: The Evolution and Adaptation of Fungi. J. Fungi.

[4] Pagano L., Mayor S. (2018). Invasive fungal infections in high-risk patients: Report from TIMM-8 2017. Future Sci. OA.

[5] Michiels L., Shameem M., Vanhoffelen E., Reséndiz-Sharpe A., Johnston S.A., Beziere N., Vande Velde G. (2025). Fungal infections in focus: Accelerating non-invasive imaging from preclinical insights to clinical breakthroughs. npj Imaging.

[6] Feys S., Carvalho A., Clancy C.J., Gangneux J.P., Hoenigl M., Lagrou K., Rijnders B.J.A., Seldeslachts L., Vanderbeke L., van de Veerdonk F.L. (2024). Influenza-associated and COVID-19-associated pulmonary aspergillosis in critically ill patients. Lancet Respir. Med..

[7] Denning D.W. (2024). Global incidence and mortality of severe fungal disease. Lancet Infect. Dis..

[8] Lai J., Wang B., Petrik M., Beziere N., Hammoud D.A. (2023). Radiotracer Development for Fungal-Specific Imaging: Past, Present, and Future. J. Infect. Dis..

[9] Zaoutis T.E., Argon J., Chu J., Berlin J.A., Walsh T.J., Feudtner C. (2005). The epidemiology and attributable outcomes of candidemia in adults and children hospitalized in the United States: A propensity analysis. Clin. Infect. Dis..

[10] Dasbach E.J., Davies G.M., Teutsch S.M. (2000). Burden of aspergillosis-related hospitalizations in the United States. Clin. Infect. Dis..

[11] Sanguinetti M., Posteraro B., Beigelman-Aubry C., Lamoth F., Dunet V., Slavin M., Richardson M.D. (2019). Diagnosis and treatment of invasive fungal infections: Looking ahead. J. Antimicrob. Chemother..

[12] Fang W., Wu J., Cheng M., Zhu X., Du M., Chen C., Liao W., Zhi K., Pan W. (2023). Diagnosis of invasive fungal infections: Challenges and recent developments. J. Biomed. Sci..

[13] Hussain K.K., Malavia D., Johnson E.M., Littlechild J., Winlove C.P., Vollmer F., Gow N.A.R. (2020). Biosensors and Diagnostics for Fungal Detection. J. Fungi.

[14] Chandrasekar P. (2010). Diagnostic challenges and recent advances in the early management of invasive fungal infections. Eur. J. Haematol..

[15] Vande Velde G., Kucharíková S., Van Dijck P., Himmelreich U. (2018). Bioluminescence imaging increases in vivo screening efficiency for antifungal activity against device-associated Candida albicans biofilms. Int. J. Antimicrob. Agents.

[16] Vanherp L., Poelmans J., Hillen A., Janbon G., Brock M., Lagrou K., Vande Velde G., Himmelreich U. (2020). The Added Value of Longitudinal Imaging for Preclinical In Vivo Efficacy Testing of Therapeutic Compounds against Cerebral Cryptococcosis. Antimicrob. Agents Chemother..

[17] Lawal I., Zeevaart J., Ebenhan T., Ankrah A., Vorster M., Kruger H.G., Govender T., Sathekge M. (2017). Metabolic Imaging of Infection. J. Nucl. Med..

[18] Sathekge M.M., Ankrah A.O., Lawal I., Vorster M. (2018). Monitoring Response to Therapy. Semin. Nucl. Med..

[19] Hammoud D.A., Clifford Lane H., Jain S.K. (2023). Molecular Imaging of Infections: Advancing the Search for the Hidden Enemy. J. Infect. Dis..

[20] Rahman W.T., Wale D.J., Viglianti B.L., Townsend D.M., Manganaro M.S., Gross M.D., Wong K.K., Rubello D. (2019). The impact of infection and inflammation in oncologic ^18^F-FDG PET/CT imaging. Biomed. Pharmacother..

[21] Glaudemans A.W., Signore A. (2010). FDG-PET/CT in infections: The imaging method of choice?. Eur. J. Nucl. Med. Mol. Imaging.

[22] Kung B.T., Seraj S.M., Zadeh M.Z., Rojulpote C., Kothekar E., Ayubcha C., Ng K.S., Ng K.K., Au-Yong T.K., Werner T.J. (2019). An update on the role of ^18^F-FDG-PET/CT in major infectious and inflammatory diseases. Am. J. Nucl. Med. Mol. Imaging.

[23] Ankrah A.O., Span L.F.R., Klein H.C., de Jong P.A., Dierckx R., Kwee T.C., Sathekge M.M., Glaudemans A. (2019). Role of FDG PET/CT in monitoring treatment response in patients with invasive fungal infections. Eur. J. Nucl. Med. Mol. Imaging.

[24] Sharma P., Mukherjee A., Karunanithi S., Bal C., Kumar R. (2014). Potential role of 18F-FDG PET/CT in patients with fungal infections. AJR Am. J. Roentgenol..

[25] Pijl J.P., Nienhuis P.H., Kwee T.C., Glaudemans A., Slart R., Gormsen L.C. (2021). Limitations and Pitfalls of FDG-PET/CT in Infection and Inflammation. Semin. Nucl. Med..

[26] Shah S., Lai J., Basuli F., Martinez-Orengo N., Patel R., Hammoud D.A. (2024). Successful development and preclinical validation of 2-Deoxy 2-[^18^F]FluoroCellobiose as an *Aspergillus*-specific PET tracer. Sci. Transl. Med..

[27] Basuli F., Shi J., Shah S., Lai J., Hammoud D.A., Swenson R.E. (2024). Fully Automated Cassette-Based Synthesis of 2-Deoxy-2-[^18^F]Fluorocellobiose Using Trasis AllInOne Module. J. Label. Compd. Radiopharm..

[28] Dahl K., Jussing E., Bylund L., Moein M.M., Samén E., Tran T. (2021). Fully automated production of the fibroblast activation protein radiotracer [^18^F]FAPI-74. J. Label. Compd. Radiopharm..

[29] Waśniowski P., Czuczejko J., Chuchra M., Wędrowski M., Marciniak D., Sobiak S., Małkowski B. (2022). Automatic Production of [^18^F]F-DOPA Using the Raytest SynChrom R&D Module. Pharmaceuticals.

[30] Poot A.J., Adamzek K.W.A., Windhorst A.D., Vosjan M., Kropf S., Wester H.J., van Dongen G., Vugts D.J. (2019). Fully Automated ^89^Zr Labeling and Purification of Antibodies. J. Nucl. Med..

[31] Al Khouri Z., Smeltzer H., Al Qura’an A., Khan M.H., Malek A.E. (2025). A Distinct Clinical Entity of Invasive Cardiac Aspergillosis: Not the Heart Valves This Time. J. Fungi.

[32] Navaratnam A.M., Al-Freah M., Cavazza A., Auzinger G. (2021). A case series of non-valvular cardiac aspergillosis in critically ill solid organ transplant and non-transplant patients and systematic review. J. Intensive Care Soc..

[33] Shah S., Turner M.L., Chen X., Ances B.M., Hammoud D.A., Tucker E.W. (2023). The Promise of Molecular Imaging: Focus on Central Nervous System Infections. J. Infect. Dis..

[34] Khan R.M.N., Ahn Y.-M., Marriner G.A., Via L.E., D’Hooge F., Seo Lee S., Yang N., Basuli F., White A.G., Tomko J.A. (2024). Distributable, metabolic PET reporting of tuberculosis. Nat. Commun..

[35] Delbeke D., Coleman R.E., Guiberteau M.J., Brown M.L., Royal H.D., Siegel B.A., Townsend D.W., Berland L.L., Parker J.A., Hubner K. (2006). Procedure Guideline for Tumor Imaging with ^18^F-FDG PET/CT 1.0. J. Nucl. Med..

[36] Kumar P., Patel C.D., Singla S., Malhotra A. (2014). Effect of duration of fasting and diet on the myocardial uptake of F-18-2-fluoro-2-deoxyglucose (F-18 FDG) at rest. Indian J. Nucl. Med..

