# Supplementary material for: Cassette-Based Automated Production of 2-Deoxy-2-[18F]fluorocellobiose on the Trasis AllInOne with Undetectable [18F]FDG Contamination
Source: Molecules. 2026 Apr 10;31(8):1260. doi: 10.3390/molecules31081260 (PMC13119221; doi:10.3390/molecules31081260)
Supplement: Supplementary file 1 [file molecules-31-01260-s001.zip › molecules-4202077-supplementary.pdf]

Supporting Information

Cassette-Based Automated Production of 2-Deoxy-2-[^{18}F]fluorocellobiose on the Trasis AllInOne with Undetectable [^{18}F]FDG Contamination

Falguni Basuli[1], Jianfeng Shi[1], Swati Shah[2], Jianhao Lai[2], Dima A. Hammoud[2], Rolf E. Swenson[1]

[1]Chemistry and Synthesis Center, National Heart, Lung, and Blood Institute, National Institutes of Health, Rockville, MD, USA

[2]Center for Infectious Disease Imaging, Radiology and Imaging Sciences, Clinical Center (CC), National Institutes of Health (NIH), Bethesda, MD, USA.

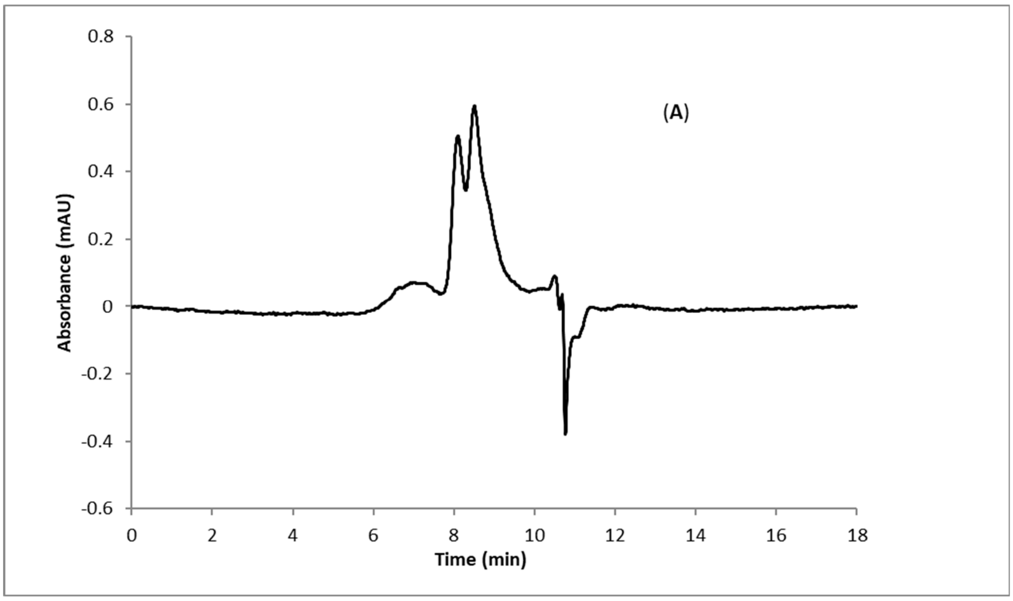
(A)
Absorbance (mAU)
0.8
0.6
0.4
0.2
0
-0.2
-0.4
-0.6
0
2
4
6
8
10
12
14
16
18
Time (min)

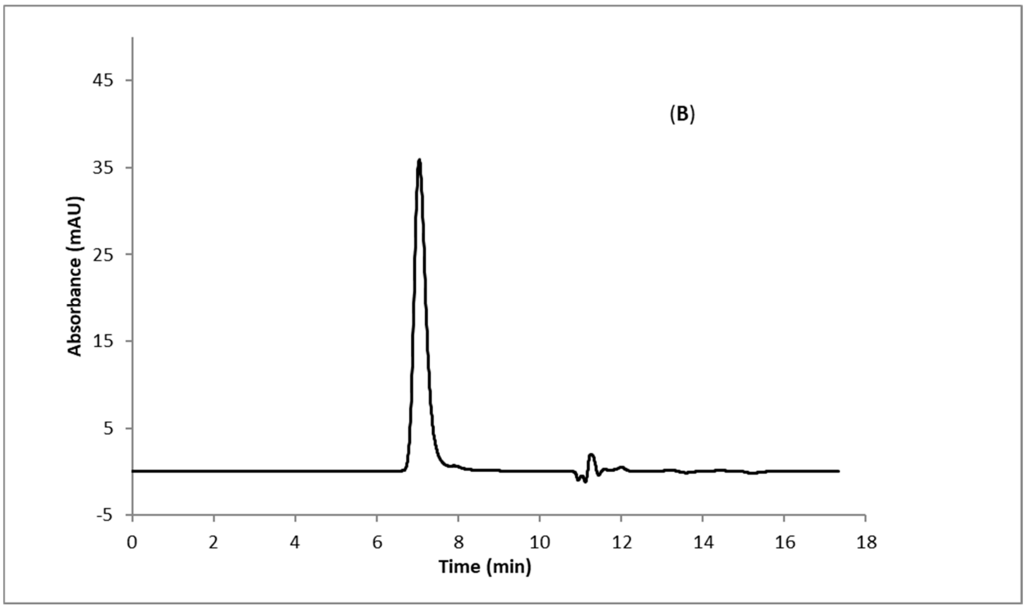
(B)
Absorbance (mAU)
45
35
25
15
5
-5
0
2
4
6
8
10
12
14
16
18
Time (min)

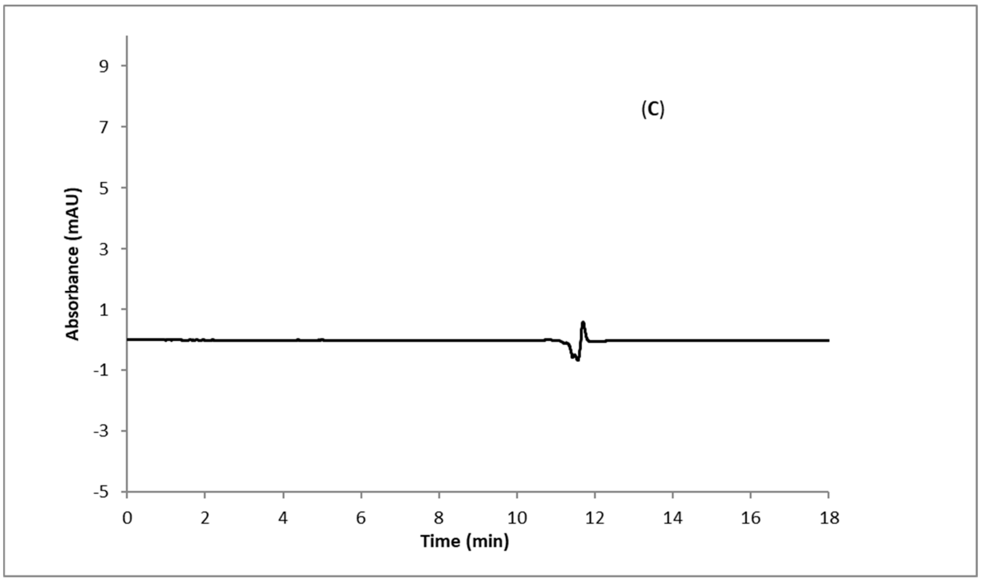

Figure S1: HPLC chromatogram A) Hexokinase (detection limit 5 ppm); B) CBP (detection limit 0.4 ppm); C) [^{18}F]FCB showing the absence of the peak for hexokinase (8 min) and CBP (7 min). HPLC conditions, Column, TSKgel SuperSW3000 (4.6 mm ID x 30 cm, 4µm); eluent, 0.1 M sodium phosphate, 0.1 M sodium sulfate, 0.05% sodium azide, 10% iso-propyl alcohol (pH 6.8), flow rate 0.35 mL/min; UV detector at 280 nm.

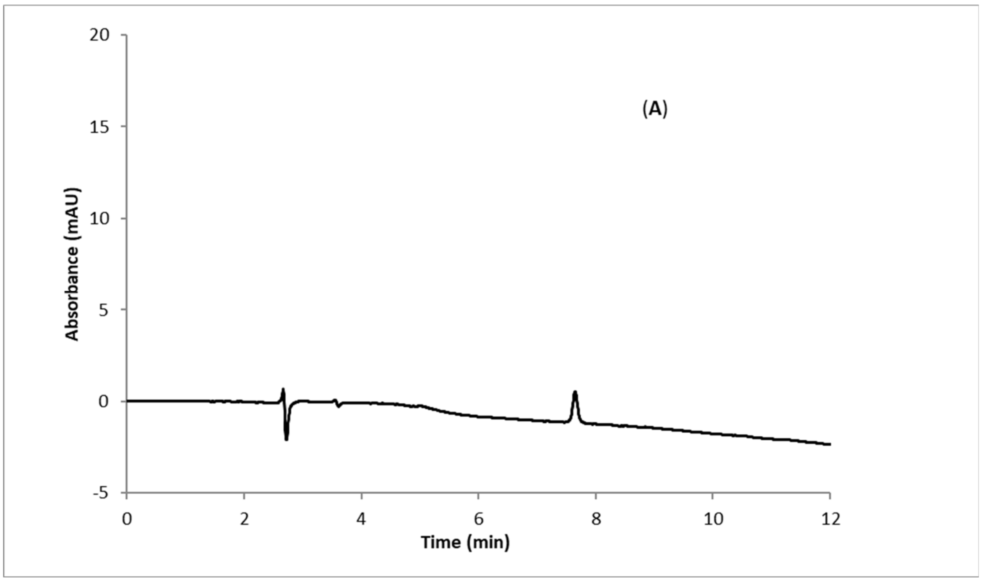

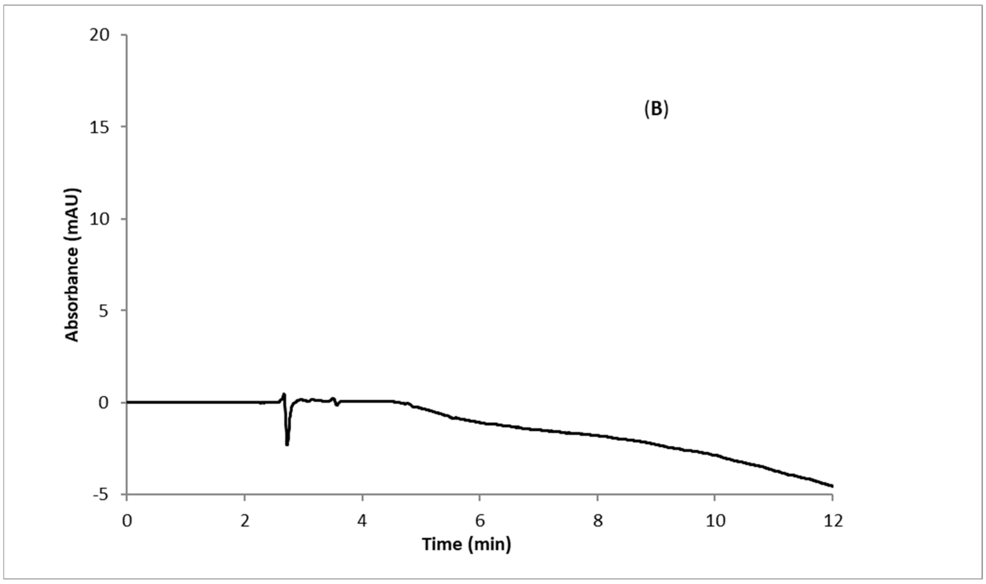

Figure S2: HPLC chromatogram A) ATP (detection limit 0.12 ppm); B) [^{18}F]FCB showing the absence of the peak for ATP (~8 min). HPLC conditions, Column, Agilent XDB C-18 (4.6 x 150 mm, 5µm); eluent, 5-100% B in A for 15 min, A = 0.1M Potassium phosphate monobasic (+ 4 mM Potassium bisulfate) in water, B = 70% A and 30% methanol, flow rate 0.5 mL/min, UV detector at 254 nm.

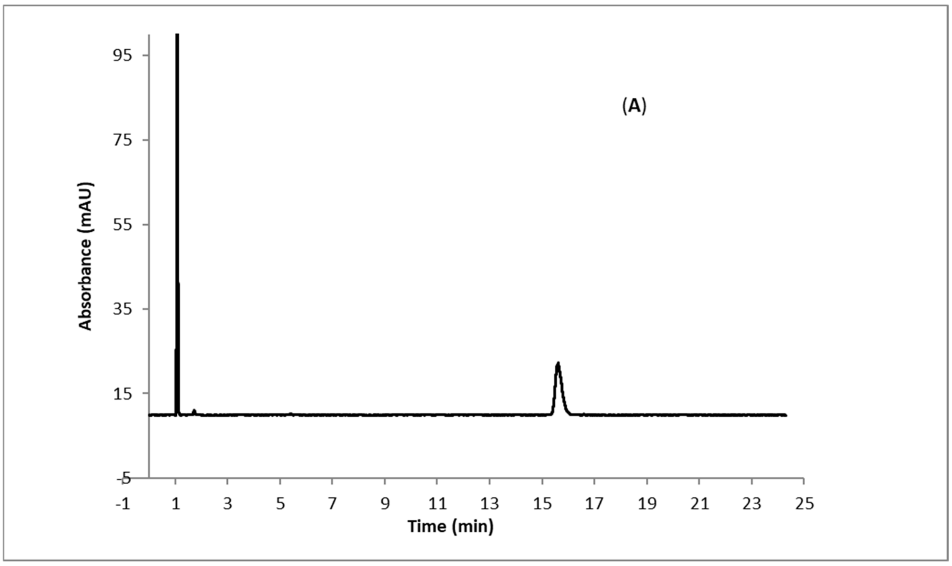

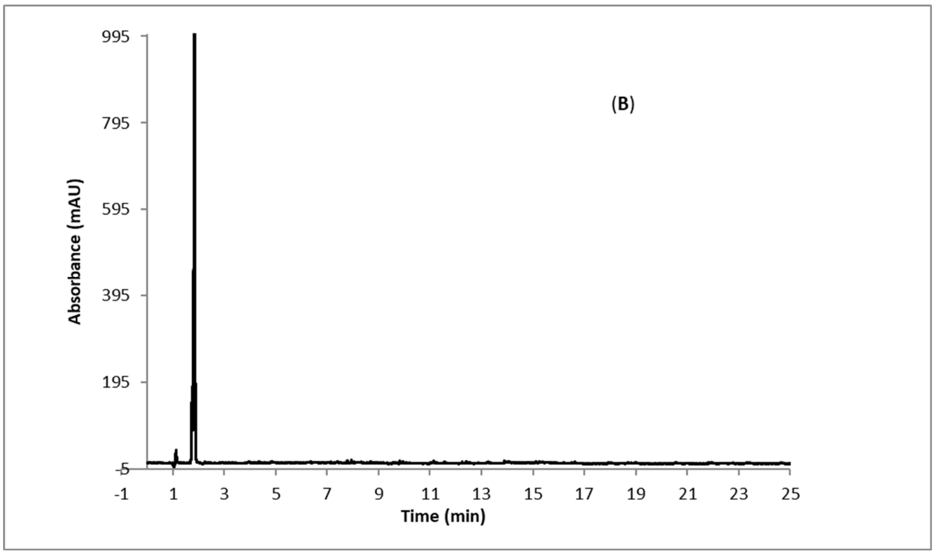

Figure S3: HPLC chromatogram A) Glc-1P (detection limit 50 ppm); B) [^{18}F]FCB showing the absence of the peak for Glc-1P (15:00 min). HPLC conditions, Column, SiELC Newcorm B (4.6 x 150 mm, 5μ, 100 A); eluent, 10% B acetonitrile in water, flow rate 1 mL/min; ELSD, evaporator temperature and nebulizer temperature 40 °C, gas flow 1.6 SLM.

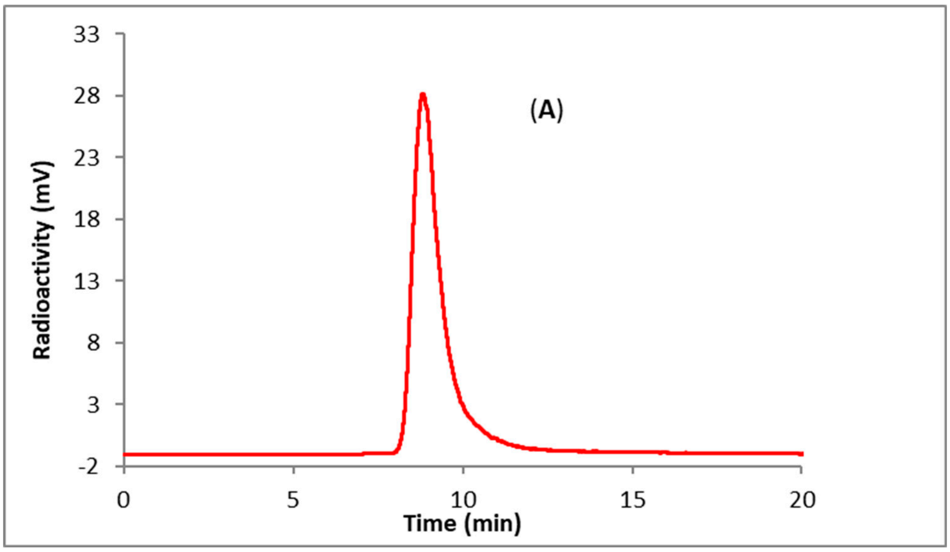

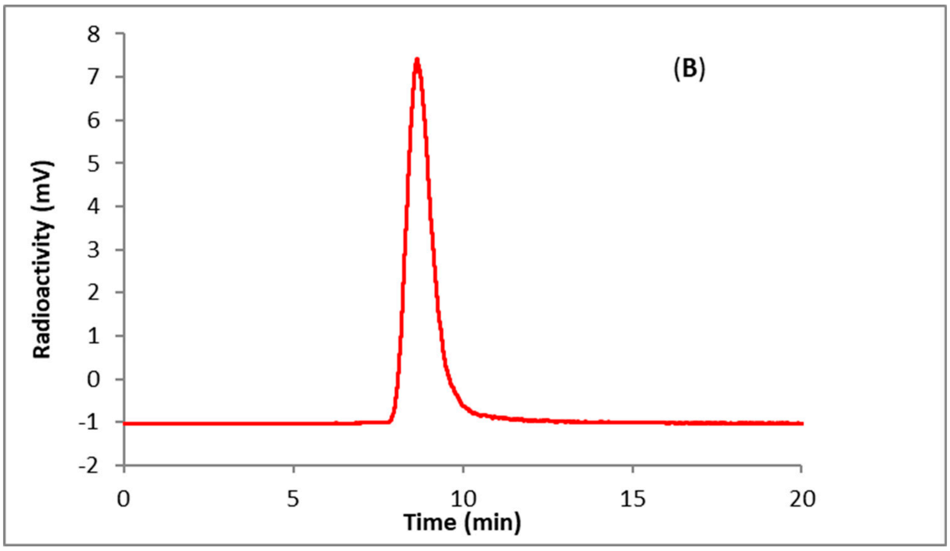

Figure S4: Stability in whole human serum A) 0 h; B) 4 h

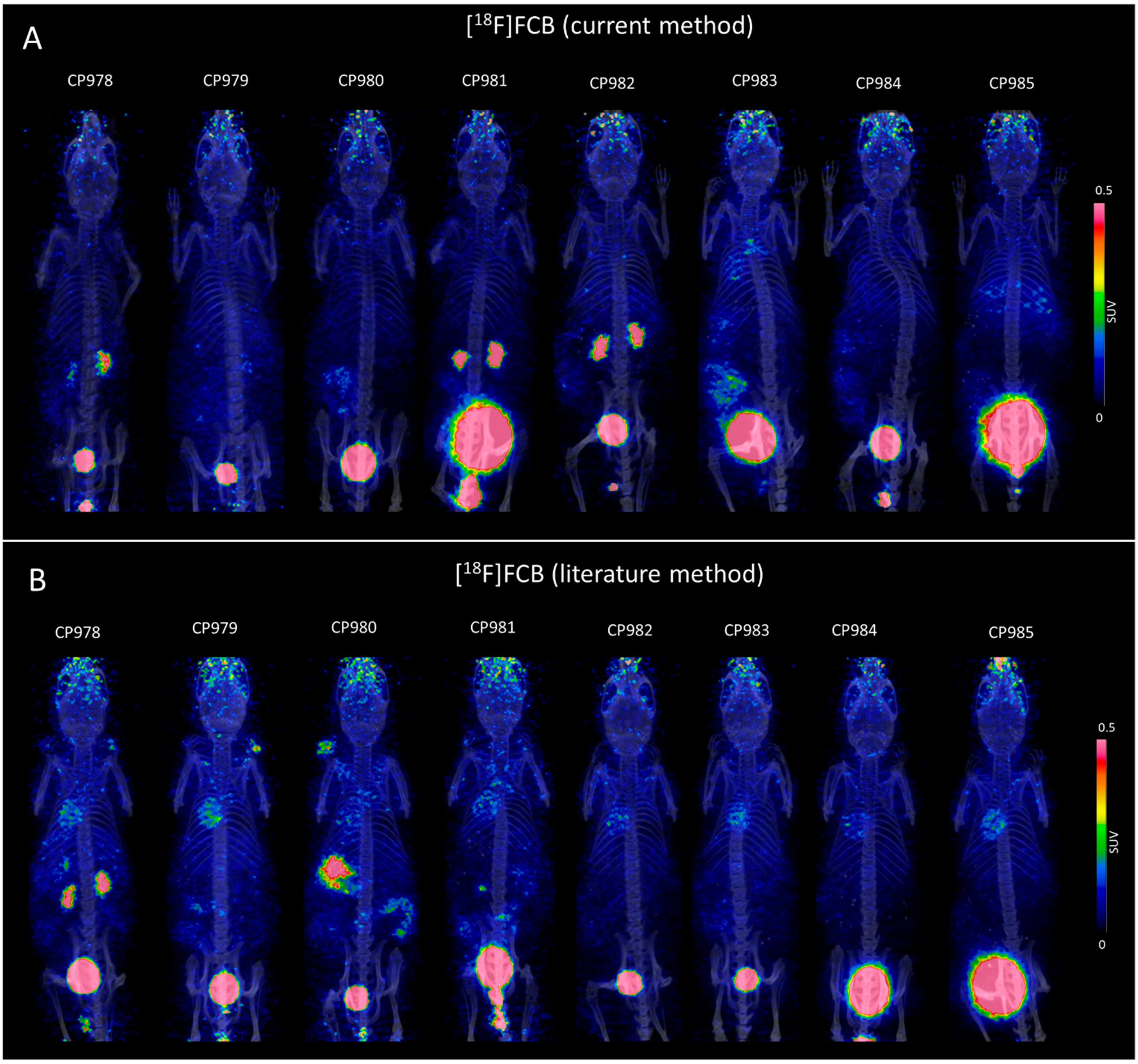

Figure S5: Maximum intensity projections of PET/CT scans obtained in the same animals using both tracer preparations (current method and literature method) in two different settings
